# Expression of AT1 and AT2 angiotensin receptors in astrocytomas is associated with poor prognosis

**DOI:** 10.1038/sj.bjc.6604431

**Published:** 2008-07-01

**Authors:** O Arrieta, B Pineda-Olvera, P Guevara-Salazar, N Hernández-Pedro, D Morales-Espinosa, T L Cerón-Lizarraga, C H González-De la Rosa, D Rembao, B Segura-Pacheco, J Sotelo

**Affiliations:** 1 Experimental Oncology Laboratory and Medical Oncology Department, Instituto Nacional de Cancerología (INCan), Tlalpan 14080, México; 2 Neuroimmunology Unit and Neuropathology Department, Instituto Nacional de Neurología y Neurocirugía (INNN), Tlalpan 14080, México; 3 Natural Sciences Department, Universidad Autónoma Metropolitana (UAM), Cuajimalpa, México

**Keywords:** angiotensin receptors, astrocytomas, angiogenesis, prognosis

## Abstract

Astrocytomas develop intense vascular proliferation, essential for tumour growth and invasiveness. Angiotensin II (ANGII) was initially described as a vasoconstrictor; recent studies have shown its participation in cellular proliferation, vascularisation, and apoptosis. We conducted a prospective study to evaluate the expression of ANGII receptors – AT1 and AT2 – and their relationship with prognosis. We studied 133 tumours from patients with diagnosis of astrocytoma who underwent surgery from 1997 to 2002. AT1 and AT2 were expressed in 52 and 44% of the tumours, respectively, when determined by both reverse transcriptase–polymerase chain reaction and immunohistochemistry. Ten per cent of low-grade astrocytomas were positive for AT1, whereas grade III and IV astrocytomas were positive in 67% (*P*<0.001). AT2 receptors were positive in 17% of low-grade astrocytomas and in 53% of high-grade astrocytomas (*P=*0.01). AT1-positive tumours showed higher cellular proliferation and vascular density. Patients with AT1-positive tumours had a lower survival rate than those with AT1-negative (*P*<0.001). No association to survival was found for AT2 in the multivariate analysis. Expression of AT1 and AT2 is associated with high grade of malignancy, increased cellular proliferation, and angiogenesis, and is thus related to poor prognosis. These findings suggest that ANGII receptors might be potential therapeutic targets for high-grade astrocytomas.

Glial tumours are the most frequent neoplasms of the central nervous system; three-quarters of these are malignant, such as anaplastic astrocytoma (AA) and glioblastoma multiforme (GM), both associated with poor prognosis (Reardon *et al*, 2006). Despite multidisciplinary therapeutic approaches, the median survival for patients with GM is approximately 12 months after diagnosis (Lopez-Gonzalez and Sotelo, 2000; Sotelo *et al*, 2006). High-grade astrocytomas (AA and GM) display high cellular proliferation and extensive angiogenesis (Tuettenberg *et al*, 2006) stimulated by the production of growth factors such as platelet-derived growth factor (PDGF), vascular endothelial growth factor (VEGF), and hepatocyte growth factor (Strugar *et al*, 1995; Moriyama *et al*, 1999; Schmidt *et al*, 1999; Arrieta *et al*, 2002). Angiotensin II (ANGII) is the main effector in the renin-angiotensin-aldosterone system (RAAS) – a powerful vasoconstrictor system – (Matsusaka and Ichikawa, 1997) and acts on two membrane-bound receptors (AT1 and AT2). Recent studies have revealed that ANGII also participates in other physiologic processes including cell growth, cell differentiation, and receptor-mediated apoptosis (Stoll *et al*, 1995; Escobar *et al*, 2004). AT1 is present in all tissues, whereas AT2 is highly expressed in pathologic conditions such as heart failure and myocardial hypertrophy (Nahmias and Strosberg, 1995; Dzau *et al*, 2001; Touyz and Berry, 2002). Some reports indicate that ANGII induces neovascularisation (Fernandez *et al*, 1985; Le Noble *et al*, 1991; Andrade *et al*, 1996) due to the upregulation of growth factors such as PDGF (Khachigian *et al*, 2000; Cook *et al*, 2002), transforming growth factor-β (Kagami *et al*, 1994; Ohta *et al*, 1994; Hamaguchi *et al*, 1999; Weigert *et al*, 2002), insulin-like growth factor-1 (Brink *et al*, 1999; Haddad *et al*, 2003), basic fibroblast growth factor (Peng *et al*, 2001), VEGF (Otani *et al*, 1998; Tamarat *et al*, 2002), and angiopoietin 2 (Otani *et al*, 2001).

A local brain RAAS loop has been described to play a role in hormone production regulation (Ganong, 1984, 1995). Regarding this, glial cells present AT1 and AT2 ANG receptor-like immunoreactivity (Fogarty and Matute, 2001). Some studies have shown that RAAS is expressed by high-grade human astrocytomas, and is able to synthesise rennin, in contrast to low-grade reactive astrocytomas which lack RAAS, thus indicating a possible relationship between endogenous intratumoral rennin and angiogenesis (Ariza *et al*, 1988; Juillerat-Jeanneret *et al*, 2004). In previous reports, the presence of AT1 was documented in rat astrocytoma cells (Fogarty *et al*, 2002). Our group has also shown that selective blockage of AT1 inhibits tumour growth, cell proliferation, and angiogenesis by inducing apoptosis in C6 rat malignant glioma (Arrieta *et al*, 2005), suggesting that AT1 plays a significant role in tumour angiogenesis and growth. In this study, we show the expression of ANGII receptors and their participation in the prognosis of patients with astrocytomas.

## Patients and methods

### Experimental design and patients

With the previous approval of the Institutional Scientific and Bioethics Committees, we conducted a prospective study at the Instituto Nacional de Neurologia y Neurocirugia (INNN) of Mexico. Tumoral tissue of 133 patients with astrocytoma who underwent surgery from 1997 to 2002 was studied. Patients who had previously received chemotherapy or radiotherapy were not included. Patients with low-grade tumours underwent surgical treatment, whereas those with high-grade tumours were treated by surgery, radiotherapy, and adjuvant chemotherapy. Patients who showed disease progression during radiotherapy were excluded. All patients included in this study were treated in a homogeneous way. Four samples of non-neoplastic human brain tissue obtained by surgery for epilepsy were used as controls. Tissue was frozen in liquid nitrogen and maintained at −70°C until AT1 and AT2 determinations were performed.

### Angiotensin II AT1 and -2 receptors

Ribonucleic acid (RNA) from tumour samples was obtained using TRIzol Reagent (Invitrogen, Carlsbad, CA, USA). To minimise the risk of contaminating DNA, total RNAs were digested with 10 U of DNase I (Boehringer Mannheim, Lewes, UK) for 15 min at 37°C. One microgram of total RNA was used for reverse transcription, which was performed with an RNA PCR Kit (Applied Biosystems, Branchburg, NJ, USA) following the manufacturer's instructions. For ANGII receptor detection, the following primers were used: sense AT1 5′-TTGCAGAGTGGGTGACAGAG-3′; antisense AT1 5′-TAGCTGAGCTTGCAGAA-3′ -3′ (393 base pairs (bp)); sense AT2 5′-TTATGGCTTTCCCACCTGAG-3′ and reverse AT2 5′-GTTGGTGAATCCCAAGAGSGA-3′ (342 bp); in a total reaction volume of 20 *μ*L. The polymerase chain reaction (PCR) conditions were: 94°C for 5 min, followed by 27 cycles at 94°C for 30 s, 60°C for 30 s, and 72°C for 60 s. The expression of the GAPDH gene was analysed as a control for the amount and integrity of the mRNA using the following primers: sense 5′-GAAGGTGAAGGTCGGAGTC-3′ antisense 5′-CAAGATGGTGATGGGATTTC-3′ (226 bp). PCR conditions were: 94°C for 5 min, followed by 27 cycles at 94°C for 30 s, 55°C for 30 s, and 72°C for 30 s.

### Immunohistochemistry for AT1 and AT2 receptors

Histological analysis was made in 5-*μ*m tissue sections that were mounted onto silanised slides. After they were deparaffinised and rehydrated, antigen was retrieved with 10 mM sodium citrate solution (pH 6.0) preheated at 80°C in a water bath, maintaining this temperature, and keeping sections in this solution for 20 min. After allowing the sections to cool to room temperature, the slides were rinsed in phosphate-buffered saline (PBS) (pH 7.4). Endogenous peroxidase activity was quenched by incubation of the tissue samples for 10 min in 3% hydrogen peroxide. Tissue samples were rinsed gently with PBS and incubated with 1% bovine serum album (BSA) for 10 min. BSA excess was eliminated and the primary antibodies were incubated for 30 min at room temperature in a moisture chamber.

Dilution of the primary antibodies against AT1 (Rabbit polyclonal IgG antibody, Santa Cruz Biotechnology Inc.) was 1:300 in BSA 1% in PBS. The polyclonal antirabbit AT1 receptor antibody (306, catalogue SC579) is specific for AT1 and is mouse, rat, and human reactive; it does not show cross-reactivity with AT2 (Lu *et al*, 1998; Ino *et al*, 2003, 2006), and AT-2 (Goat polyclonal IgG Santa Cruz Biotechnology Inc.) was 1 : 300 in BSA 1% in PBS. One tissue section was used for each antibody. After washing with PBS, binding of primary antibodies was induced by incubation for 20 min with labelled streptavidin biotin System Link (LSAB) (DAKO Corporation; Carpinteria, CA, USA) and LSAB+ HRP (Streptavidin HRP Kit, DAKO Corporation). The slides were rinsed with PBS and exposed to the diaminobenzidine chromogen for 5 min. After washing with PBS and counter-staining with haematoxylin, the slides were dehydrated by means of graduated alcohols and xylol, and mounted with Poly-mount (Polysciences Inc., Warrington, PA, USA). The slides were observed by light microscopy. Ten high-power fields (40 ×) were analysed in each specimen. Samples that showed absence of immunoreactivity were considered negative and those with some percentage of immunoreactivity were considered positive. The percentage of AT-positive tumour cells was estimated and graded into one of three categories: 1=low (<35% of positive cells), 2=intermediate (35–75% positive cells), or 3=high (>75% positive cells) (Takeda and Kondo, 2001). Observations were made by a pathologist in a blinded manner.

### Determination of vascular density, mitotic, and cell proliferation indexes

Mitotic and cell proliferation indexes as well as vascular density were determined by IHC as previously described (Arrieta *et al*, 2002). We analysed only 30 tumours owing to insufficient biopsy material. Briefly, a small tissue sample was fixed in 10% formalin, and 5-*μ*m slices were obtained. Standard haematoxylin-eosin stain was used for histological confirmation of diagnosis. Additional samples were used for IHC with the avidin–biotin–peroxidase complex, and were counterstained with haematoxylin. Samples were incubated for 45 min at room temperature either with rabbit antihuman factor VIII-related antigen (DAKO Corporation) as marker for vascular endothelial cells, or with mouse antiproliferation cell nuclear antigen (PCNA; DAKO corporation) as marker for cellular synthesis phase. A pathologist (DR) counted the number of capillaries positive for factor VIII and the number of cell nuclei positive for PCNA per microscopic field at × 40 magnification in 10 different fields, in a blinded manner.

### Statistical analysis

For descriptive purposes, continuous variables were summarised as arithmetic means, medians, and standard errors (s.e.), categorical variables as proportions with 95% confidence intervals (95% CI). Inferential comparisons were performed by Student's *t-* or Mann–Whitney *U*-tests according to distribution of the data (normal and non-normal) as determined by the Kolmogorov–Smirnov test. *χ*^2^ or Fisher's exact test was used to assess significance between categorical variables. Statistical significance was determined as *P*<0.05 with a two-sided test. Statistically significant and borderline significant variables (*P*<0.1) were included in the multivariate logistic regression analysis. Overall survival time was measured from day of surgery to date of death of the patient and was analysed with the Kaplan–Meier technique, whereas comparisons among subgroups were performed with the log-rank test. For survival curves analysis, all variables were dichotomised. Adjustment for potential confounders was made by multivariate regression analysis. SPSS software package version 10 (SPSS Inc., Chicago, IL, USA) was employed to analyse the data.

## Results

### Patient characteristics and histopathologic diagnosis

Of 133 tumours, low-grade astrocytomas comprised 28% (14% grade I, and 14% grade II), whereas 72% were high-grade astrocytomas (17% grade III, and 55% grade IV). Median age of the patients was 47±1.6 years and mean follow-up time was 9.4±2.2 months (range: 0.1–111 months). Performance status score (ECOG) was 1 in 29%, 2 in 26%, and 3 in 45% of patients.

### Expression of AT1 and AT2 receptors

**Figure 1:**
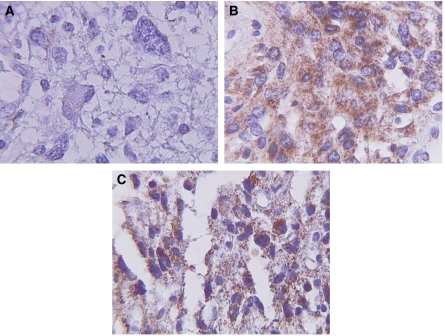
Immunoreactivity for AT receptors in high-grade astrocytoma: (**A**) negative, (**B**) AT1 positive, and (**C**) AT2 positive.

**Figure 2:**
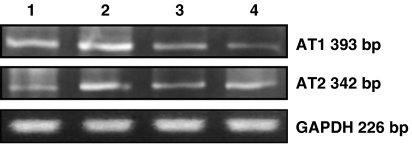
Determination of AT1 and AT2 mRNAs by RT–PCR performed in three high-grade astrocytoma surgical samples; lane 1 corresponds to positive control; lanes 2, 3, and 4 correspond to different surgical samples.

Angiotensin II receptors expression was analysed by reverse transcriptase (RT)–PCR in 133 tumours and analysed by IHC in only 112 because of insufficient tissue samples. AT1/AT2 was not detected in sections of human brain tissues obtained by surgery for epilepsy (data not shown). The expression of both receptors was detected in high-grade astrocytomas (Figure 1B and C). IHC showed a cytoplasmatic as well as discrete membrane and nuclear localisation of AT1 (Figure 1B). Fifty-two per cent were positive for AT1 (95% CI, 43.3–60.4), whereas 44% (95% CI, 35–52) were positive for AT2 by both methods. There were no tumours displaying high-intensity staining (>75% of cells). There was a significant correlation (0.73; 95% CI 0.65–0.81; *P*<0.001) between the presence of AT1 and AT2 (*P*<0.001), demonstrating that 45% of tumours expressed both receptors. Moreover, AT1/AT2 expression was detected by RT–PCR (Figure 2). All cases showed expression of GAPDH. Three representative cases are shown in Figure 2. Correlation between both methods was 0.93 (95% CI 0.88–0.97; *P*<0.001). There were no samples with negative RT–PCR and positive IHC results.

### AT1 expression and its clinicopathologic correlation

Table 1 shows the correlation between AT1 expression obtained by both RT–PCR and IHC according to malignancy grade. The expression of AT1 was found more frequently in high-grade tumours (grades 3 and 4) when compared with low-grade tumours (67 *vs* 10%, respectively; *P*<0.0001). Other associated factors with AT1 expression included: age >47 years (67 *vs* 43%; *P=*0.019) and poor performance status (65 *vs* 39%; *P=*0.03). In multivariate analysis, the only significant factors associated with the presence of AT1 were age (Hazard's ratio (HR) 3.2 (95% CI, 1.04–10.2); *P=*0.04) and histological grade (HR 13.4 (95% CI, 3.2–55); *P*<0.0001). AT1-positive tumours showed higher mitotic index (3.8±0.1 *vs* 2.8±0.1; *P*<0.001), higher proliferation index (66.8±0.54 *vs* 51.6±2.3; *P*<0.001), and higher vascular density (16±0.4 *vs* 13.6±0.5; *P*<0.002) when compared to AT1-negative tumours. There was no correlation between IHC-staining intensity and grade of malignancy.

### AT2 expression and its clinicopathologic correlation

Table 1 shows the correlation of AT2 expression – obtained by both RT–PCR and IHC – and grade of malignancy. High-grade tumours had a greater expression of AT2 than low-grade tumours (53 *vs* 17%, respectively; *P=*0.001). An additional associated factor was poor functional status (56 *vs* 30%; *P=*0.04). In the multivariate analysis, only the grade of malignancy was associated with the presence of AT2 (HR 3.4; 95% CI, 1.08–10.86; *P=*0.036).

Tumours that expressed this receptor showed a higher mitotic index (3.95±0.17 *vs* 3.15±0.1; *P=*0.001), higher proliferation index (66.4±0.6 *vs* 58.1±2.4; *P=*0.004), and higher vascular density (16.7±0.39 *vs* 14.06±0.4; *P*<0.001) when compared to AT2-negative tumours. There was no correlation between the intensity of IHC staining and grade of malignancy.

### Survival of patients with malignant astrocytomas

**Figure 3:**
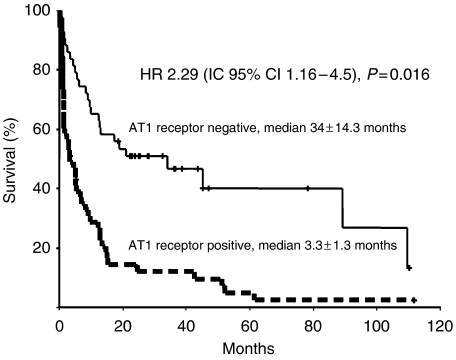
Overall survival and AT1 expression.

Median overall survival for all patients was 9.7±2.0 months. Table 2 shows survival-associated factors in both the univariate and the multivariate analyses. Survival-associated factors in the univariate analysis were histological grade (I/II *vs* III/IV; *P*<0.001), age (⩽47 *vs* >47 years; *P*<0.001), performance status (ECOG ⩽1 *vs* ⩾2; *P*<0.001), and mitotic index (⩽3.3 *vs* >3.3; *P=*0.007), predictive factors previously reported. Interestingly, patients with AT1-positive tumours (IHC- and RT–PCR positive) showed a lower survival rate (3.3±1.3 *vs* 34±14.3; *P*<0.0001). The presence of AT2 was also associated with a decrease in overall survival (3.3±1.6 *vs* 12.8±3; *P=*0.006). There was no association between the intensity of IHC staining for both AT1 and AT2 and survival. In the multivariate analysis, statistically significant factors for poor prognosis were: diagnosis of high-grade astrocytoma (*P=*0.05), poor performance status (*P*<0.001), high mitotic index (*P=*0.03), and AT1 expression (*P=*0.01, (Figure 3)).

## Discussion

Previous reports have described the expression of both RAAS and AT1 in normal brain tissue as well as their possible participation in cerebral vascular regulation (Ganong, 1984). Likewise, the presence of AT1 and AT2 in C6 glioma cells has been described, showing that their blockage can inhibit tumoral growth and angiogenesis (Rivera *et al*, 2001; Fogarty *et al*, 2002; Arrieta *et al*, 2005). Recent reports describe the local expression of many components of RAAS in several human neoplasms, among these human GM (Deshayes and Nahmias, 2005). We show the presence of AT1 and AT2 in 52 and 44% of human astrocytomas, respectively; therefore, it will be important to evaluate the cellular function of this type of molecules in the near future. Although there was a significant correlation (0.93) between both methods, four tumours were positive for the expression of AT receptors by RT–RCR but were negative by IHC. This is probably due to the higher sensibility of the former method or because the tumour is expressing the AT-receptors’ mRNA, but not the protein at the membrane level. The IHC shows expression of AT1 and AT2 in tumoral cells, discarding the fact that the results obtained by RT–PCR are due to the presence of AT1 and AT2 in vascular brain tissue. We observed the localisation of AT1 receptor within the nucleus, membrane, and cytoplasm. These observations concur with previous reports in which nuclear ANGII receptors have been demonstrated in brain neurons, hepatocytes, and human embryonic kidney (HEK-293T) (Booz *et al*, 1992; Lu *et al*, 1998; Lee *et al*, 2004), thus suggesting AT2-induced nuclear sequestration of the AT1 receptor.

Expression of AT1 and AT2, as well as other RAAS components in cells from human astrocytomas, was variable, possibly related to clonal heterogeneity (Juillerat-Jeanneret *et al*, 2004). However, we found that the presence of AT1 and AT2 was associated with the grade of malignancy. Similar findings have been reported with the expression of ANGII in normal brain tissue, in low-grade tumours, and in high-grade tumours (Juillerat-Jeanneret *et al*, 2004).

The presence of ANGII receptors in AA and GM and their correlation with mitotic and proliferation indexes, as well as with vascular density, suggest that both AT1 and AT2 play a role in the regulation of several tissular processes such as angiogenesis, cell proliferation, invasiveness, and apoptosis (Escobar *et al*, 2004), either through the stimulation or the inhibition of RAAS components locally synthesised by the tumour (Juillerat-Jeanneret *et al*, 2004). In this matter, we found that there is co-expression of AT1 and AT2 in malignant tumours, suggesting a possible relation between both receptors. It is known that the stimulation of AT2 can inhibit AT1 activation pathways, leading to growth inhibition. The presence of both receptors in the same tumour suggests that selective blockage of one receptor could increase the effect of the other (Arrieta *et al*, 2005). It is possible that AT1 inhibition produces disequilibrium in the AT1/AT2 stimulation, which in turn favours AT2 stimulation, thus opening the possibility of the pathway leading mainly to apoptosis. There are other endogenous peptide hormones of the RAAS, such as AT1-5 and AT1-7, which could regulate proliferation processes in neoplastic cells; it has been demonstrated that AT1-7 inhibits proliferation and induces apoptosis of human lung cancer cells through its receptor *MAS* (Gallagher and Tallant 2004). Further studies should be performed to determine the presence of these peptides and their receptors in human gliomas to establish their influence over their cellular proliferation and behaviour.

We also found an independent association between AT1 expression and vascular density. Previous reports have shown that the stimulation of this receptor augments VEGF expression in various tumoral and non-tumoral tissues and that its blockage inhibits VEGF expression in gliomas and other tissues (Chua *et al*, 1998; Tamarat *et al*, 2002; Arrieta *et al*, 2005; Imai *et al*, 2007). In addition, AT1 selective blockage inhibits cell proliferation and neovascularisation in experimental C6 rat glioma (Rivera *et al*, 2001; Arrieta *et al*, 2005), which is known to express AT1 (Rivera *et al*, 2001; Fogarty *et al*, 2002). Similarly, experimental AT1 blockage has shown growth inhibition in other neoplasms such as melanoma (Egami *et al*, 2003), lung carcinoma (Fujita *et al*, 2005), prostate adenocarcinoma (Uemura *et al*, 2005), renal cancer (Miyajima *et al*, 2002), and pancreatic cancer (Fujimoto *et al*, 2001). Although other growth factors have been involved in ANGII-induced tumoral angiogenesis, it appears that VEGF plays a crucial role in this matter. It has been shown that when murine Lewis lung carcinoma (LLC) cells – which express AT1 – are implanted subcutaneously into wild-type mice, they develop tumours that exhibit intense angiogenesis and induction of VEGF. However, when LLC cells are implanted in AT1a gene-deficient (AT1a−/−) mice, tumour growth and tumour-associated angiogenesis are reduced, with reduced expression of VEGF (Imai *et al*, 2007). Coincidentally, VEGF is rather important in GM pathophysiology: a critical difference between a low- and high-grade astrocytoma is the increase in VEGF-induced vascular density (Yao *et al*, 2001). This growth factor is essential in the malignant progression of gliomas (Godard *et al*, 2003; Karcher *et al*, 2006). The fact that AT1 is present in high-grade astrocytomas and in patients >47 years of age suggests that AT1 and AT2 could be associated with the progression of malignancy in secondary malignant astrocytomas.

In our study, we found that histological grade, ECOG performance status, and age were factors associated with survival in the multivariate analysis. These factors have previously shown their prognostic importance in patients with high-grade astrocytomas treated with radiotherapy and adjuvant chemotherapy, and have been included in the recursive partitioning analysis developed to compare the survival categories to obtain homogeneous subsets of patients. Some studies have reported an interaction between age and genetic alterations (such as TP53 mutations and EGFR amplification) in GM, suggesting that tumorigenic pathways to GM vary according to the patient's age (Batchelor *et al*, 2004). The complex interactions between age and genetic alterations could explain the association between the former and the AT1 expression. Furthermore, given the age dependency of these genetic effects, it will be intriguing to analyse the genetic expression patterns in younger and older patients to ascertain AT1 and AT2 genetic pathways in astrocytomas.

This is the first report documenting the association between the presence of AT1/AT2 and poor prognosis in patients with high-grade astrocytoma. This study demonstrates that AT1 expression is an independent survival-related factor, suggesting that this receptor plays an essential role in the pathophysiology of high-grade astrocytomas.

A limitation of our study is the difference in the survival rates compared to those reported in the literature. Patients with low-grade astrocytoma have a survival average of 7–10 years in contrast to our population, which showed a survival median of 3.4 years. In the same manner, patients with high-grade astrocytomas, treated with chemotherapy and concomitant radiotherapy and posteriorly with adjuvant temozolamide, have a median survival of 12.1–14.6 months (Mirimanoff *et al*, 2006), in contrast to the patients in our study who displayed a median survival of 7 months. These disparities could be accounted for by: (1) the nature of the studies (observational *vs* controlled studies), and (2) differences in the treatment schema. All patients with high-grade astrocytomas in our study were treated with radiotherapy and adjuvant chemotherapy based on carmustine.

In conclusion, our study demonstrates that AT1 and AT2 are strongly expressed in high-grade astrocytomas, and it is the first report that shows an association with poor prognosis. These preliminary results suggest that these receptors are attractive therapeutic targets.

## Figures and Tables

**Table 1:** Proportion of negative and positive tumours to AT1 and AT2 expression by RT–PCR and IHC according to malignancy grade

	AT1*		AT2**	
**Tumour grade**	**Negative (%)**	**Positive (%)**	**Negative (%)**	**Positive (%)**
Grade 1	12 (86)	2 (14)	12 (86)	2 (14)
Grade 2	14 (93)	1 (7)	12 (80)	3 (20)
Grade 3	5 (28)	13 (72)	7 (39)	11 (61)
Grade 4	21 (34)	40 (66)	30 (49)	31 (51)
Total	52 (48)	56 (52)	61 (56.5)	47(43.5)

Abbreviation: RT–PCR=reverse transcriptase–polymerase chain reaction.

*P<0.001, ^**^P=0.008.

**Table 2:** Survival associated factors

	**Univariate analysis**		**Multivariate analysis**	
**Factor**	**Months±s.e.**	***P*-value**	**HR (CI 95%)**	***P*-value**
*Age (years)*				
⩽47	24.5±9.11	<0.001	1.79 (1.035–3.103)	0.037
>47	5.5±1.3			
				
*Gender*				
Male	12.6±1.9	0.262		
Female	5.4±2.6			
				
*ECOG*				
⩽1	61.4±28.2	<0.001	1.46 (1.06–2.025)	0.021
⩾2	5.5±1.6			
				
*Grade*				
I/II	42.6±22.1	<0.001	1.2 (1.0–1.5)	0.05
III/IV	7.03±1.51			
				
*MI*				
⩽3.3	33.9±10.8	0.007	5.6 (1.181–26.7)	0.03
>3.3	5.4±1.52			
				
*IPCNA*				
⩽65	14.9±27	0.14		
>65	6.6±4			
				
*VD*				
⩽15.9	12.5±0.7	0.48		
>15.9	9.2±5			
				
*AT1*				
Positive	33.9±14.1	<0.001	2.056 (1.189–3.5)	0.01
Negative	3.3±1.4			
				
*AT2*				
Positive	12.8±3.01	0.006	0.725 (0.5–1.64)	0.7
Negative	3.3±1.58			

Abbreviations: CI= confidence interval; HR=Hazard's ratio; IPCNA=index of proliferation cell nuclear antigen; MI=mitotic index; s.e.=standard error; VD=vascular density.
